# Nitridation effect on lithium iron phosphate cathode for rechargeable batteries†

**DOI:** 10.1039/d1ra07574h

**Published:** 2022-01-28

**Authors:** Sergio Federico Mayer, Cristina de la Calle, María Teresa Fernández-Díaz, José Manuel Amarilla, José Antonio Alonso

**Affiliations:** Instituto de Ciencia de Materiales de Madrid (ICMM), Consejo Superior de Investigaciones Científicas (CSIC) Sor Juana Inés de la Cruz 3 E-28049 Madrid Spain s.mayer@csic.es; Institut Laue Langevin (ILL) BP 156X F-38042 Grenoble France

## Abstract

A novel oxynitride Li_0.94_FePO_3.84_N_0.16_ with olivine structure (space group *Pnma*, no. 62) has been synthesized by heating a parent LiFePO_4_ precursor obtained by citrate chemistry in flowing ammonia at 650 °C. The polycrystalline sample has been characterized by X-ray and neutron powder diffraction (NPD), elemental and thermal analysis, scanning electron microscopy (SEM) and electrochemical measurements. Based on the existing contrast between the scattering lengths of the N and O species, a Rietveld refinement of the structure from NPD data revealed that N preferentially occupies the O2 positions, as likely required to fulfil the bonding power of N ions. The refined crystallographic formula implies an oxidation state of 2.2+ for Fe cations. The differential thermal analysis, in still air, shows a strong exothermic peak at 520–540 °C due to the combustion of C contents, which are embedding the olivine particles, as observed by SEM. The electrochemical measurements suggest a better performance for the nitrided sample relative to the unnitrided LiFePO_4_ material, as far as capacity and cyclability are concerned. A bond-valence energy landscape study reveals a decrease in the percolation activation energy of about 6% upon nitridation, concomitant with the better electrochemical properties of the oxynitride compound. Additionally, ceramic samples prepared under NH_3_ flow could be obtained as pure and well-crystallized olivine phases at milder temperatures (650 °C) than those usually described in literature.

## Abstract 2

A better-performing oxynitride with LFP olivine structure as an appealing intercalation cathode for lithium-ion batteries.

## Graphical abstract

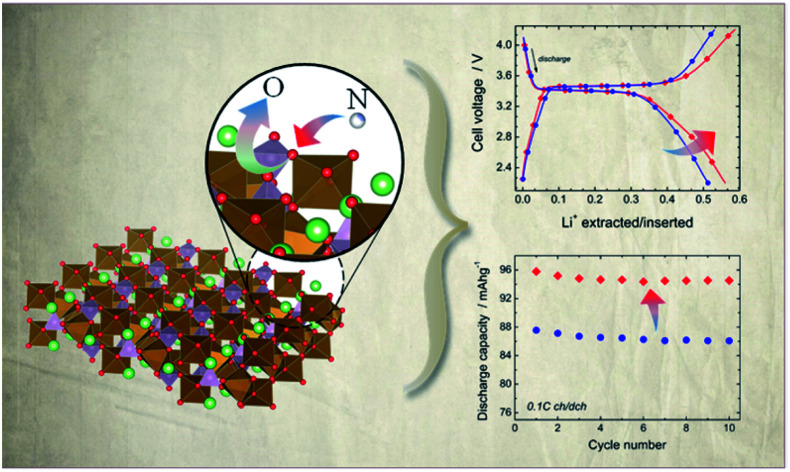

## Introduction

In order to store chemical energy, rechargeable lithium batteries^1^ (LIBs) are of particular interest since they have a high degree of portability together with high conversion efficiency. The LIB is indeed a top representation of solid-state chemistry. The first-generation LIB has a graphite negative electrode (anode), a non-aqueous liquid electrolyte, and a positive electrode (cathode) formed from layered LiCoO_2_. During the charging process, lithium ions are de-intercalated from the layered LiCoO_2_ host, travel across the electrolyte, and are intercalated between the graphite layers in the anode. Discharge reverses this process.^2^ Cost, environment, and shelf-life considerations have also driven a search for alternative cathode systems. Over the past 40 years, intensive research on transition metal oxides suitable as hosts for a rapid insertion/extraction of alkali ions has led to the development of different materials in which parameters such as structure, chemistry, morphology, synthesis method, and texture (powdered, nanoparticulated, nanostructured or mesoporous phases) have been tuned. Among this rather broad variety of materials, NASICON-related framework structures based on the Ti^4+^/Ti^3+^ or Fe^3+^/Fe^2+^ redox couples and presenting tetrahedral polyanions (XO_4_)^2−^ or (XO_4_)^3−^, with X = Mo, W, S, P, and Si, have been largely studied.^3–17^ Besides possessing high redox potentials and promising Li^+^ transport, polyanion frameworks can exhibit outstanding stability for daily-use batteries,^18–21^ hence appropriate for applications where safety and durability are a concern. Treatments over the polyanions, such as fluorination, have also received much attention due to their appealing properties; for instance, the incorporation of fluoride fosters a charge difference and variations in the lattice parameters along with an increase in the redox potential at which lithium insertion takes place.^22,23^ More recently, studies over boron-doped phosphates also presented enhanced electrochemical properties and cyclability up to a certain B content, above which oxygen vacancies are generated and the performance is hindered.^24^

Olivine LiFePO_4_ (LFP) is an appealing cathode because of its low cost, high thermal and chemical stability, and inexpensive and environmentally friendly composition.^25,26^ LFP has a lower working voltage (3.45 V *vs.* Li^+^/Li) compared to other positive electrodes, safely above the HOMO level of organic electrolytes such as liquid-carbonates,^26^ making it more stable towards electrolytes and gaining higher electrochemical stability. The orthophosphate (PO_4_)^3−^ anion has a relatively strong P–O covalent bond that lowers the top of the oxygen p-band well below the active redox energy, allowing full de-lithiation without evolving molecular O_2_ and making it more stable when compared to other cathodic materials, such as cobaltites.^16,27–29^ The intercalation mechanism involves a two-phase equilibrium between FePO_4_ and LiFePO_4_. Upon deintercalation of Li^+^-ions from a particle of LiFePO_4_, a FePO_4_ crust is formed around the particle. The phase boundary between this crust and the LiFePO_4_ core moves throughout the particle along with the continued removal of lithium.^28,30,31^

If the diffusion of lithium ions and/or electrons is considered to be the bottleneck of the de-/intercalation reactions kinetics of micro-sized LFP-based cathodes, nanostructured approaches could lower the kinetic barriers by reducing the diffusion distances. Studies^32^ showed that LFP-nanoparticles exhibited a wider range of non-stoichiometry phases than the micro-sized ones, and this non-stoichiometry may in part account for the enhanced rate of Li^+^ availability.^33^ In the last decades, novel low-temperature approaches towards the hydrothermal/solvothermal synthesis of phosphate or silicate electrode materials have been developed by means of (1) “latent bases”, also called “forced hydrolysis”, tuning the reaction pH with a basic medium created by the temperature-driven decomposition of urea into NH_3_ and CO_2_ upon hydrolysis at 90 °C,^34^ and (2) making use of ionic liquids instead of water as the reacting media.^35^ Ionic liquids present both additional advantages of having exceptional solvent properties and exhibiting high thermal stability and negligible volatility, so the use of an autoclave is not mandatory. The richness and versatility of such approaches towards the preparation of materials having controlled size and shape was reviewed and highlighted by Recham *et al.*^36^

In many cases, LiFePO_4_ samples have been nanopainted with a conducting coat to improve the electronic conductivity. For this purpose, carbon is mostly used, since its high proportion of sp^2^ linkages ensures good electronic transport between the particles.^37^ Many types of carbon-containing precursors have been used as carbon coating sources to prepare LFP/C composites.^27,38–40^ Research has shown that the quality of the carbon coating depends on the thickness of the coating,^41^ the content of sp^2^ carbon,^37^ the morphology,^42^*etc.*

In search of enhanced electronic and ionic conductivity of LFP cathodes, the benefits of O^2−^ for N^3−^ substitution in polyoxoanionic electrode materials has been scarcely studied, mainly from a theoretical point of view.^29,43–45^ First-principle calculations showed that doping with nitrogen favours the Li de-/intercalation processes from/into LFP, as doping leads to lower Li-diffusion activation energies and improved Li-ion transport properties.^43,45^ To the best of our knowledge, only two empirical LFP N-doping experiments were conducted to date,^29,46^ where superficial nitrogen-for-oxygen substitution treatments are performed. From the former studies, it is concluded that partial substitution of O^2−^ by N^3−^ in LFP olivine can promote the coexistence of Fe^2+^/Fe^3+^ couple by increasing the electrical conductivity. Besides, nitridation offers the possibility of utilizing the redox chemistry of transition metal ions plus that of the substituting p-element. Electron energy loss spectroscopy (EELS) measurements in Li_7−*x*_MnN_4_ (0 < *x* < 1.2), used as electrode in Li-batteries, confirmed that N^3−^ anions play an important role in maintaining the charge balance during Li^+^-extraction in ternary nitrides.^47^ To date, no bulk N-doped LFP phase has been conceived, so this promising research area has yet to be explored.

Motivated by these facts, we synthesized and characterized an N-doped LiFePO_4_ material, which operates on the Fe^2+^ → Fe^3+^ redox couple and is prepared in its reduced, lithiated form, so it can be used as an active cathode material in Li-ion batteries. The main purpose of this work is to provide evidence on the possibility of introducing N-anions in the O sublattice of a bulk cathode material and to shed some light on its performance. Replacement of [PO_4_]^3−^ by [PO_4−*x*_N_*x*_]^(3+*x*)−^ anions opens up the search for new oxynitride families of host lattice structures and compositions; the charge balance and the dimensionality concerning the pristine phosphate could also be changed. The present paper explores a simple and straightforward synthesis method to introduce nitrogen on the framework in a stable way and compares the electrochemical properties of the obtained N-substituted or nitrided polyoxoanionic compounds regarding the pristine LFP. It is important to remark that its implementation as cathode material, with the pertinent carbon coating, was not optimized here. We thus focus our results in the relative performance of our new nitride LFP cathode with respect to a pristine sample; a cathode preparation involving carbon nanocoating should, by extension, enable capacities of about 10% more than state-of-the-art LFP materials.

## Experimental

Our lithium intercalation compounds, suitable as active materials in positive electrodes (cathodes) in LIBs, have been synthesized in the form of micro- and sub-micrometric particles by ceramic and by sol–gel approaches, respectively, and then treated by thermal nitridation in flowing ammonia. In total, four samples were prepared, two by following a ceramic synthesis, and two by the sol–gel procedure. Ceramic procedure: (a) LiFePO_4_ compound was prepared by solid-state reaction of stoichiometric amounts of FeC_2_O_4_, (NH_4_)_2_HPO_4_ and Li_2_CO_3_ intimately ground under inert atmosphere (N_2_) to prevent the formation of Fe^3+^ compounds as impurities. This first sample was prepared by a two-step 12 h calcination process at 350 and 800 °C with an intermediate grinding process. Then, the sample was heated for 3 hours at 650 °C in flowing ammonia (99.9%). The obtained compound was labelled as LFP-Ceram1. (b) The starting ceramic mixture was directly heated in the furnace at 650 °C for 3 hours in flowing ammonia; the sample was labelled as LFP-Ceram2. Sol–gel route: stoichiometric amounts of analytical grade FeC_2_O_4_, (NH_4_)_2_HPO_4_ and Li_2_CO_3_ were dissolved in citric acid with few droplets of HNO_3_ 65% solution. The solution was slowly evaporated, leading to the formation of an organic resin, later dried at 120 °C. Afterwards, two different procedures were followed. (a) The dry-resin was heated in N_2_ atmosphere at 350 °C for 3 h, then finely ground, and later heated at 650 °C for 10 hours. The unnitrided sample was labelled as U-LFP. (b) The black U-LFP powder was nitrided by heating for 3 hours at 650 °C in ammonia flux, and then labelled as N-LFP, standing for the nitrided sample.

The obtained solids were characterized by X-ray diffraction (XRD) using a Bruker-AXS D8 diffractometer (40 kV, 30 mA) in Bragg–Brentano reflection geometry with Cu Kα radiation (*λ* = 1.5418 Å) and a solid-state detector (SOL-X). This detector has excellent energy resolution and is the ideal means of removing unwanted background features (*i.e.*, fluorescence linked to Fe atoms). Data were collected in steps of 0.05° (2*θ*) over the range 10–120° with a counting time of 4 s per step. Neutron powder diffraction (NPD) diagrams were collected at the Institute Laue Langevin, Grenoble (France) at room temperature (RT) at the D2B diffractometer with a *λ* = 1.594 Å wavelength. The XRD and NPD patterns were analysed by the Rietveld method^48^ using the FULLPROF refinement program.^49^ A pseudo-Voigt function was chosen to generate the line shape of the diffraction peaks. The neutron coherent scattering lengths for Li, Fe, P, O and N were: −1.90, 9.45, 5.13, 5.803, 9.36 fm, respectively.

Chemical quantitative analyses of the N and C content were performed immediately after preparing the samples, and ten days later, using a PerkinElmer 2400 series II CHNS/O Elemental Analyzer. Based on the classical Pregl-Dumas method, samples were combusted in a pure oxygen environment, and the resultant combustion gases were analysed in an automated fashion.

Differential thermal analysis (DTA) and thermogravimetric (TG) curves were simultaneously obtained in a Thermal Analysis ATD/DSC/TG model Q600 TA Instruments; in the temperature range from 25 to 900 °C. The temperatures of the peaks were measured with an accuracy of ±1 °C. The sample and reference were situated in platinum crucibles and the analyses were carried out in still air at 10 °C min^−1^ heating/cooling rate.

The morphology of the products prepared by the different synthetic routes used was examined from scanning electron microscopy (SEM). Secondary electron imaging and back-scattered electrons were performed in an SEM HITACHI S-3000N with an Energy Dispersive X-ray analyser from Oxford Instruments, INCAx-sight. A sputter coater SC502 was used for gold coating.

The comparative study of the electrochemical properties of N-doped and precursor LiFePO_4_ olivine was evaluated using two-electrode lithium half-cells. Although we are aware that controlled amounts of carbon painting have a critical impact on the final electrochemical performance of the cathode,^41,50^ here we used a standard preparation method for relative measurement of the electrochemical properties of both nitrided and unnitrided cathodes, rather than obtaining the best-announced performance. Hence, performance determinations should be interpreted only relatively. Positive electrode composites were prepared from the olivine powders (∼10 mg, or 85 wt%), MMM Super P carbon black (10 wt%) and Fluka polyvinylidene fluoride (PVDF, 5 wt%). The three powder components were dispersed in 1-methyl-2-pyrrolidinone as fugitive solvent and stirred overnight. The slurry was then dried at 80 °C overnight, and then cylindrical pellets of 12 mm diameter and 300 μm thickness were obtained by cold pressing at RT at 370 MPa. The negative electrode was a 0.6 mm thick lithium foil, which also operates as reference electrode. The electrodes were separated by a Whatman BSF80 paper soaked in the electrolyte, *i.e.*, a 1 M solution of LiPF_6_ in ethylene carbonate and dimethyl carbonate (EC : DMC, 1 : 1 v/v) as supplied by UBE Europe GmbH. The components were assembled into a CR2032 coin-cell within an argon glove box in which water content was kept below 1 ppm. Cells were galvanostatically cycled at RT in the voltage range from 2.2 to 4.2 V at 0.1C charge/discharge rates with an Arbin-BT4 battery system (1C = 170 mA g^−1^). After 10 cycles, the cells were discharged at increasing rates from 0.2C (34 mA g^−1^) to 5C (850 mA g^−1^) with a fixed charge rate of 0.5C. Five cycles were performed for every rate in the voltage range 2.2 to 4.2 V.

## Results and discussion

### Synthesis and elemental chemical analysis (N and C)

The elemental nitrogen content analysis shows a lack of this element in the LFP-Ceram1 and U-LFP samples, whereas the one performed on the LFP-Ceram2 sample exhibits a 0.75 wt% and 0.72 wt% of N content (immediately after synthesis and 10 days later, respectively), and 1.47 and 1.45 wt% in the N-LFP sample for the same respective studies. No significant loss of nitrogen was observed over time. Although it is hard to assess the amount of N that is actually bonded to the carbonaceous matrix, the nitrogen content in the N-LFP sample act as a theoretical maximum of the N that can be found substituting oxygen of the LFP structure. Therefore, the LFP-Ceram2 yields a N amount of 0.08, while N-LFP presents a maximum of 0.33 N atoms per formula unit, in the cited experimental conditions. Attempts to increase the N contents in LFP-Ceram2 (involving a further thermal treatment at 650 °C under NH_3_) lead to a progressive loss of N. From these observations we conclude that it is virtually impossible to nitride a pre-shaped olivine phase obtained by ceramic methods (*i.e.*, LFP-Ceram1) and that the sol–gel route yields the most nitrided phases. In addition, we have also conducted measurements of the C contents. For the samples obtained by the sol–gel route, namely U-LFP and N-LFP, the amount of C was 50.3% and 50.6%, respectively. In fact, in an inert or reducing atmosphere, there is no combustion of the organic precursor resin; therefore, an important amount of carbon is present in both samples in the nanostructured form, which sizably would contribute to the electronic conductivity of the samples but rather hinder the active Fe(iii) availability and the reversible capacity of the cathode. The amount of C was considered in the interpretation of electrochemical measurements. Small amounts of C, around 0.5 wt%, were detected in the samples obtained by ceramic methods, likely coming from the decomposition of the Fe oxalate.

### Room temperature X-ray (XRPD) and neutron (NPD) powder diffraction

**Fig. 1:**
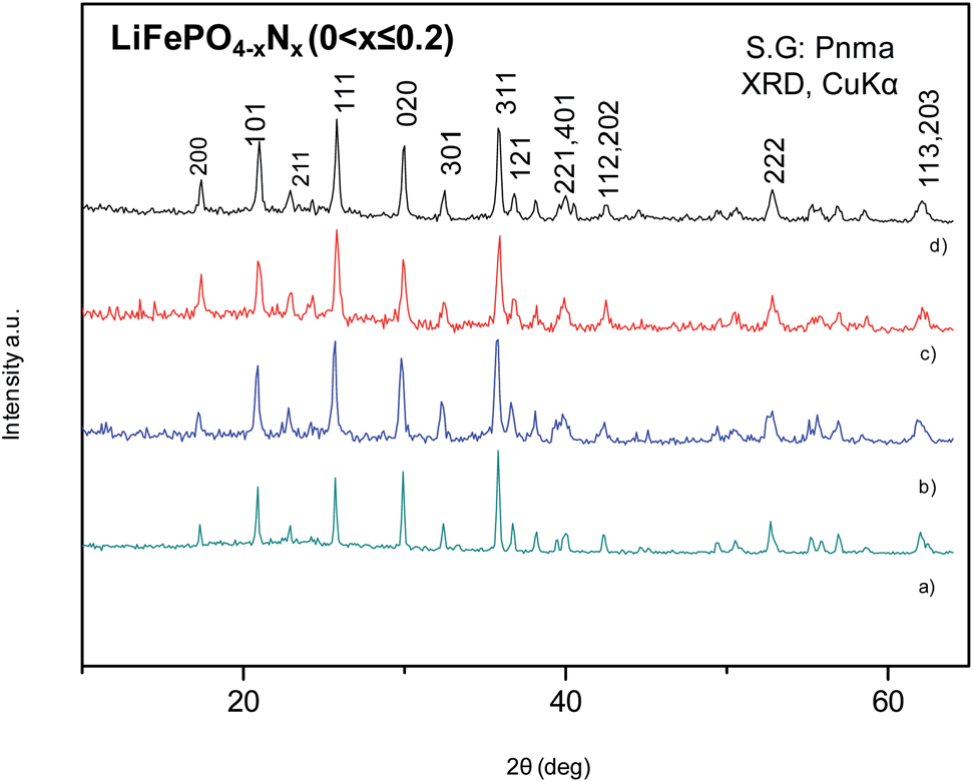
XRD patterns (Cu Kα) of (a) LFP-Ceram1, (b) LFP-Ceram2, prepared by ceramic procedures, and (c) U-LFP and (d) N-LFP prepared by citrate routes. All of them can be indexed in an orthorhombic unit cell, *Pnma* space group (no. 62).

**Fig. 2:**
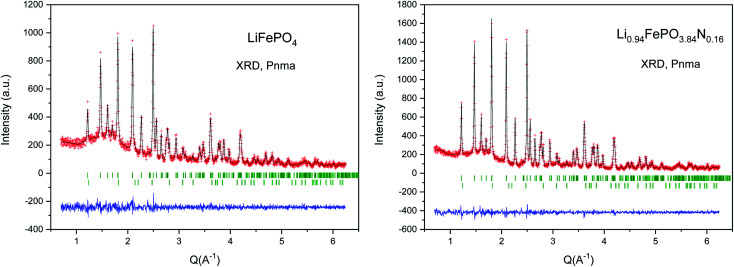
Rietveld plots for U-LFP (left) and N-LFP (right), prepared by citrate techniques, from XRD data. The two series of Bragg markers correspond to the main phase and minor amounts of Fe_2_P (barringerite).

The XRPD patterns of the LFP-Ceram1, LFP-Ceram2, U-LFP and N-LFP samples, prepared under different synthesis routes, show well-defined reflections corresponding to an orthorhombic unit cell, as displayed in Fig. 1. Even though the synthesis routes and chemistry are different, the XRPD patterns of the final products look very similar and correspond to single olivine phases. It is noteworthy that, among the four different compounds, the LiFePO_4_-Ceram2 sample was obtained as a pure and well-crystallized phase, starting from a ceramic mixture and only treated for 3 h at 650 °C in ammonia flux; this gas stream enables the formation of the olivine framework at fairly moderate temperatures. It is known that the nitridation temperature domain has an upper limit; in fact, according to some authors, heating up pristine LFP in a reducing atmosphere at 800 °C or above causes partial decomposition of the phosphate with subsequent loss of phosphorous element.^51,52^ This precludes the use of ‘standard’ (high temperatures and long times) ceramic methods under ammonia flux. We have established here that it is possible to synthesize olivine phases in an ammonia atmosphere from ceramic mixtures at temperatures below the limit of partial decomposition of phosphates and in short synthesis times. Nevertheless, we should recall that temperatures as high as 800 °C and times up to 24 h are needed to prepare LiFePO_4_ by solid-state reactions in an inert atmosphere,^16^ thus indicating the benefits of ammonia atmosphere in lowering the synthesis temperature by at least 150 °C, as compared to classical ceramic approaches in inert conditions. The ammonia media promotes this decrease of the synthesis temperature, through the use of ‘latent bases’. In this novel method, NH_3_ is provided directly as flowing gas. The lattice parameters of LFP-Ceram1, LFP-Ceram2, U-LFP and N-LFP are listed in Table 1; these values were obtained by Rietveld refinement of the structures from XRD data. The four compounds are defined in the *Pnma* (no. 62) space group. The products prepared by the citrate chemistry route exhibit an increase in the unit–cell parameters relative to those obtained by the ceramic methods. The presence of N in LFP-Ceram2 and N-LFP is also revealed by a tiny increase of the lattice parameters with respect to LFP-Ceram1 and U-LFP compounds, respectively. The good agreement between experimental and calculated XRD intensities is illustrated in Fig. 2 for nitrided and unnitrided samples obtained by sol–gel methods, namely N-LFP and U-LFP, respectively. Small quantities (about 1%) of Fe_2_P impurity phase were detected; this barringerite phase^53^ is usually formed on heating at temperatures around 700 °C in reducing atmospheres.^52,54^ This impurity was included and quantified in the Rietveld refinements as a second minor phase.

**Table 1:** Lattice parameters (Å) and volume (Å^3^) of LFP-Ceram1, LFP-Ceram2, U-LFP and N-LFP, determined by Rietveld refinement of the crystal structure in the orthorhombic *Pnma* space group (no. 62), *Z* = 4, from XRD data

	LFP-Ceram1	LFP-Ceram2	U-LFP	N-LFP
N content/f.u.	—	0.1	—	0.2
Synth. cond.	Sol. st. chemistry	Sol. st. chem. + NH_3_	Citrate chemistry	Citrate chem. + NH_3_
a	10.2897(5)	10.315(3)	10.321(1)	10.3294(8)
b	5.9918(3)	6.003(2)	6.0066(7)	6.0078(5)
c	4.6966(2)	4.696(1)	4.6853(6)	4.6949(4)
V	289.56(2)	290.78(1)	290.46(6)	291.35(4)

**Fig. 3:**
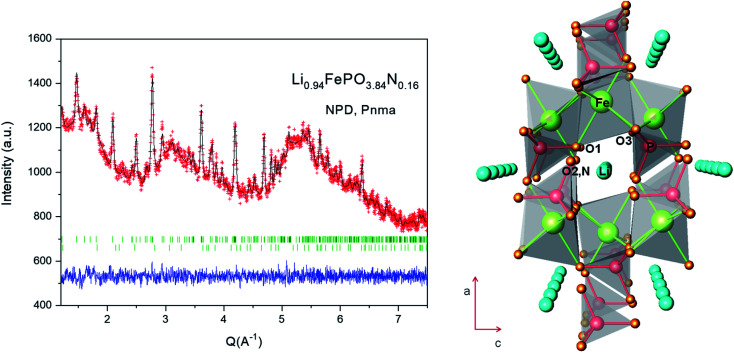
(a) Rietveld plot of Li_0.94_FePO_3.84_N_0.16_ from NPD data at RT. The second series of Bragg markers correspond to minor impurities of Fe_2_P. The large background is due to the presence of amorphous carbon. (b) View of the crystal structure of Li_0.94_FePO_3.84_N_0.16_, highlighting the presence of tunnels along the [010] direction favouring the reversible removal of Li^+^ ions.

Neutron powder diffraction experiments (NPD) were performed in order to identify the distribution of nitrogen atoms in the framework, species impossible to be differentiated from O atoms by X-ray diffraction. The use of neutron diffraction to determine the site occupancy of oxygen and nitrogen is possible because of the large difference in their coherent neutron scattering lengths (9.36 and 5.803 fm for N and O, respectively). It has been shown elsewhere^55^ that nitrogen N^3−^ may exist in [PO]_4_ tetrahedra as doubly coordinated (–N=) and triply coordinated (–N <svg xmlns="http://www.w3.org/2000/svg" version="1.0" width="10.400000pt" height="16.000000pt" viewBox="0 0 10.400000 16.000000" preserveAspectRatio="xMidYMid meet"><metadata> Created by potrace 1.16, written by Peter Selinger 2001-2019 </metadata><g transform="translate(1.000000,15.000000) scale(0.011667,-0.011667)" fill="currentColor" stroke="none"><path d="M480 1160 l0 -40 -40 0 -40 0 0 -40 0 -40 -40 0 -40 0 0 -40 0 -40 -40 0 -40 0 0 -40 0 -40 -40 0 -40 0 0 -40 0 -40 -40 0 -40 0 0 -80 0 -80 40 0 40 0 0 40 0 40 40 0 40 0 0 40 0 40 40 0 40 0 0 40 0 40 40 0 40 0 0 40 0 40 40 0 40 0 0 40 0 40 40 0 40 0 0 40 0 40 40 0 40 0 0 40 0 40 -80 0 -80 0 0 -40z M80 480 l0 -80 40 0 40 0 0 -40 0 -40 40 0 40 0 0 -40 0 -40 40 0 40 0 0 -40 0 -40 40 0 40 0 0 -40 0 -40 40 0 40 0 0 -40 0 -40 80 0 80 0 0 40 0 40 -40 0 -40 0 0 40 0 40 -40 0 -40 0 0 40 0 40 -40 0 -40 0 0 40 0 40 -40 0 -40 0 0 40 0 40 -40 0 -40 0 0 40 0 40 -40 0 -40 0 0 40 0 40 -40 0 -40 0 0 -80z"/></g></svg> ) atoms, respectively bonded to two or three phosphorus atoms. They replace bridging (P–O–P) as well as non-bridging (O=P) oxygen atoms, inducing a more covalent and cross-linked structure. The collected NPD pattern of N-LFP presents a large background scattering due to the significant presence of amorphous carbon, in spite of which the crystal structure was successfully refined. In LiFePO_4_, most atoms (Fe, P, O1 and O2) are distributed in the special position 4*c*.^56^ Li can be found in an inversion centre at 4*a* site, while O3 is in the 8*d* general Wyckoff position. The atomic positions reported by Rousse *et.al*.^57^ were used as the initial model for the Rietveld refinement. The refinement of mixed O/N occupancy factors for O1, O2 and O3 positions unambiguously led to localizing N at O2 sites, as nitrogen was rejected from O1 and O3 positions. The occupancy of the lithium species was also refined and resulted to be stoichiometric within three standard deviations. About 1% of Fe_2_P impurity (barringerite) was included as a second phase in the refinement. The final occupancy factors, together with other atomic parameters after the full refinement of the crystal structure, are included in Table 2. Fig. 3a shows the goodness of the fit. Table 3 summarizes main bond distances for N-LFP after the refinement from NPD data. LiFePO_4_ belongs to the olivine family and presents a triphylite FePO_4_ type-structure. Olivine can be described with the AB_2_O_4_ formula, where the tetrahedral and octahedral cavities of an HCP oxygen network are respectively occupied by the A and B atoms. In the LiFePO_4_ parent phase, both Li and Fe atoms are distributed in octahedral sites in a very different fashion. Li^+^ ions establish one-dimensional chains that allow percolation parallel to the [010] orthorhombic direction (see Fig. 3b); each heavily-distorted FeO_6_ octahedron shares corners with four PO_4_ tetrahedra and with four additional FeO_6_ octahedra. In this structure, tunnels along which Li^+^-ions can percolate are identified in the [010] direction, as highlighted in Fig. 3b. In the unnitrided sample,^57^ Li is connected to two O1, two O2 and two O3 oxygen atoms.

**Table 2:** Structural parameters for Li_0.94_FePO_3.84_N_0.16_ (N-LFP) after the Rietveld refinement of NPD data at RT. Space group *Pnma*; *a* = 10.323(1), *b* = 6.0050(9), *c* = 4.6930(6) Å, *V* = 290.93(7) Å^3^^a^

Atom	Site	*x*	*y*	*z*	*f*_occ_	*B*_iso_ (Å^2^)
Li	4*a*	0	0	0	0.94(2)	1.71(9)
Fe	4*c*	0.2828(7)	0.25	0.982(2)	1.0	1.21(1)
P	4*c*	0.096(1)	0.25	0.414(2)	1.0	0.40(2)
O1	4*c*	0.098(1)	0.25	0.743(2)	1.0	0.57(1)
O2	4*c*	0.459(1)	0.25	0.214(3)	0.84(4)	0.57(1)
N	4*c*	0.459(1)	0.25	0.214(3)	0.16(4)	0.57(1)
O3	8*d*	0.164(1)	0.0467(1)	0.2849(17)	1.0	0.57(1)

a Reliability factors: *R*_p_ = 1.41%, *R*_wp_ = 1.76%, *R*_exp_ = 1.83%, *χ*^2^ = 0.932, *R*_Bragg_ = 5.32%.

**Table 3:** Selected bond distances (Å) in Li_0.94_FePO_3.84_N_0.16_ (N-LFP) from NPD data at RT

**FeO_6_ octahedron**	
Fe–	O1: 2.214(16)
	O2: 2.118(15)
	O3: 2.081(7) × 2

**LiO_6_ octahedron**	
Li–	O1: 2.175(10) × 2
	O2: 2.058(9) × 2
	O3: 2.176(10) × 2

**PO_4_ tetrahedron**	
P–	O1: 1.544(17)
	O2: 1.540(17)
	O3: 1.533(9) × 2

The partial replacement of O2 by N is surprising because the Li–(O2, N) distances are the shortest among the Li–O bond lengths. This is probably required to fulfil the bonding power of N ions. A calculation of the bond valence by Brown's model suggests that N is still underbonded, with a valence of −2.54(8). Since O and N are sharing the same crystallographic sites, diffraction methods yield average bond lengths (M–O =M–N), and the local M–N distances (which cannot be determined in a diffraction experiment) are probably much closer to those expected for N^3−^. The partial occupancy of O2 sites by N maximizes the interactions with Li since its presence narrows the tunnels along [010] directions across which Li diffuses during the deintercalation process.

### Bond-valence energy landscape, Li-ion percolation

A bond-valence energy landscape (BVEL) analysis, based on the bond-valence theory,^58^ let us determine from reliable structural data the activation energy required to achieve the Li^+^ ion percolation across the structure. We observed that upon N doping, the diffusion activation energy (*E*_a_) of the Li^+^ ion in the 1-dimensional *b* direction decreases from 0.96 eV for U-LFP, to 0.91 eV for the nitrided sample (N-LFP), a total reduction of 6.1%. This likely occurs owing to an increase in the covalency of the framework, as N has a lower Pauling electronegativity (3.04) than oxygen (3.44).^59^

Although the rigidity of the covalent framework tends to increase upon the increase of the covalency, the higher anionic charge and covalent radius of the N regarding the O species (−3 and 1.32 Å *vs.* −2 and 1.21 Å,^60^ respectively) gives nitrogen higher polarizability, and hence, establishes a stronger link to P atoms by sharing their electrons. This effect lowers the polyanionic affinity to the electropositive diffusive species, namely Li^+^ ions, hence percolating easier throughout the channelled structure of the LFP.

**Fig. 4:**
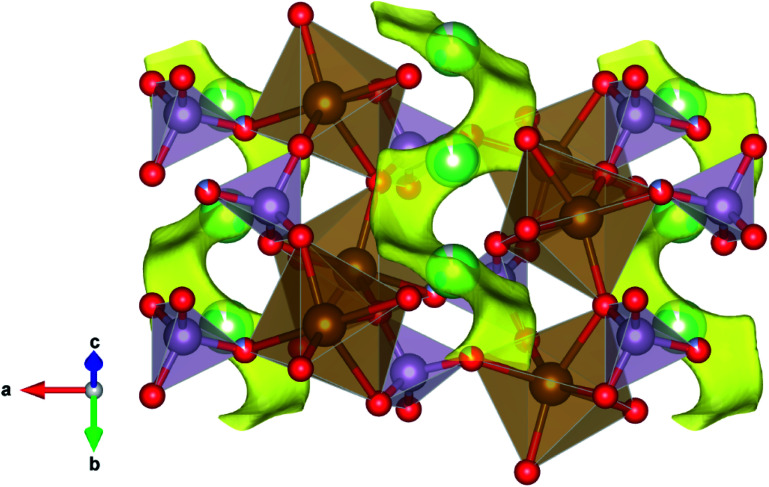
View of the percolation pathway for N-LFP along the *b* direction, corresponding to the one-dimensional percolation path of lowest activation energy, *E*_a*b*_ = 0.91 eV. Li1 species is located within the percolation pathway, here presented as a yellow isosurface.

A view of the described percolation mechanism generated by the BVEL method for the N-LFP sample is presented in Fig. 4, where the Li ions percolate in the *b* direction only. An animated detail of the structure and the percolation path is included as ESI.†

The anions involved in the percolation process are located in bond-site energy wells and possess a distinctive bond site energy (SE).^61^ In cathodes, it is theoretically and experimentally demonstrated that the higher the SE, the lower the potential these can achieve for a given setup within a LIB.^62^ As N-LFP presents a lower SE than U-LFP (−4.73 eV and −4.55 eV, respectively), it is expected that N-LFP exhibits a slightly higher cell potential. This, together with the fact that N-LFP experiences an increase in the Li-ion percolation volume of the cell of about 6% (from 9.79% for U-LFP to 10.37% for N-LFP), makes it an appealing candidate as cathode in LFP-type LIBs.

### Thermal stability of the phases

**Fig. 5:**
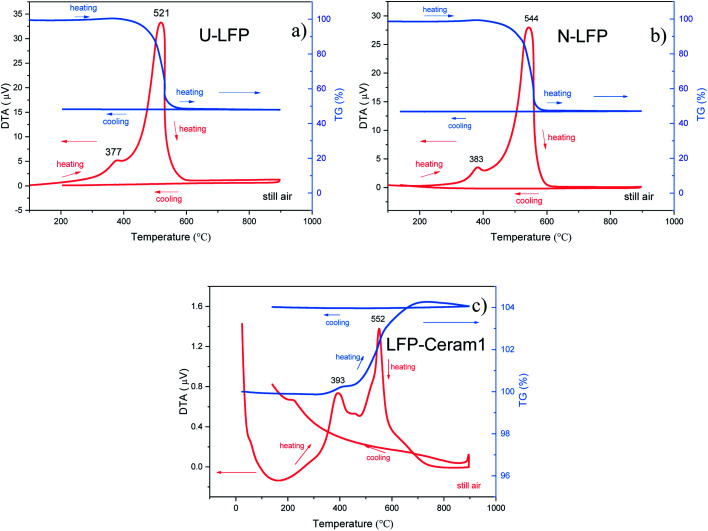
Thermal analysis curves (TG, right axis; DTA, left axis) in air of (a) unnitrided U-LFP, (b) nitrided N-LFP, (c) LFP-ceram1.

We evaluated the thermal stability of our LFP materials by DTA-TG analysis in the RT-900 °C temperature range, in air. The thermal analysis curves are displayed in Fig. 5. These phases exhibited no obvious endothermic or exothermic effects below 350 °C. This means that the studied compounds have higher thermal stability than other cathode materials such as LiCoO_2_, LiNiO_2_ or LiMn_2_O_4_.^18–20^ In addition, U-LFP and N-LFP DTA curves show two consecutive exothermic peaks. The first peak present in each sample, located at 377 and 383 °C, respectively, are associated with a small gain of weight, between 0.5 and 1.0 wt%, observed on the TG profiles around 380 °C. The second, strong exothermic peaks, at 521 °C for U-LFP and 544 °C for N-LFP, are coupled to a large loss of weight of roughly 50 wt%, observed beyond 410 °C in both cases (Fig. 5a and b). This corresponds to the combustion of the carbon contents of the samples. To compare with the carbon-free compounds, a DTA-TG analysis was performed on LFP-Ceram1 in the same analysis conditions. Fig. 5c shows the DTA curve with the two exothermic peaks but much less intense, and the TG profile reveals a gain of weight of ∼0.3% at 390 °C and another one of 4% at about 500 °C. These thermal transitions are irreversible, as shown on the cooling stage, and correspond to the decomposition of the samples.

After the thermal treatment in air, the XRD patterns of the three samples show new diffraction peaks, which can be indexed as the oxidation products of LFP: Li_3_Fe_2_(PO_4_)_3_ (PDF file: 43-0526) and Fe_2_O_3_. In light of these results, we can confirm that, upon heating in air, the LFP materials show thermal stability up to 350 °C. Beyond this temperature, in all three cases, an oxidation reaction that takes place in two stages occurs, with the consequent formation of two oxidized products. In addition, for U-LFP and N-LFP, there is a combustion of the carbon contents, with a substantial loss of weight that masks the weight gain corresponding to the second oxidation process of the sample itself. Noticeably, the weight loss observed in the thermograms of the U-LFP and N-LFP samples indeed corresponds to the amount of C determined by elemental chemical analysis.

### Topographic and local composition studies by SEM

**Fig. 6:**
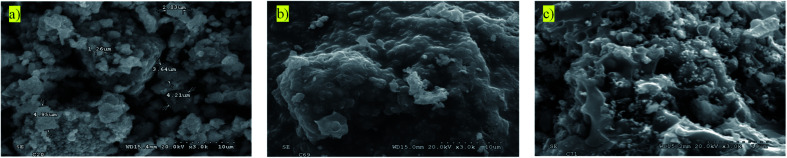
Scanning electron microscopy images for (a) LFP-Ceram1, (b) U-LFP and (c) N-LFP.

In order to understand the surface structure of the LFP phases, scanning electron microscopy (SEM) experiments have been conducted. Morphological characterization and local energy-dispersive X-ray (EDX) chemical analyses were performed on LFP-Ceram1, U-LFP and N-LFP samples. The SEM images of the first material (Fig. 6a) reveal a heterogeneous crystallite size distribution from 1 to 5 μm. U-LFP and N-LFP images (Fig. 6b and c) display LFP grains entirely embedded within the carbonous structure, and varying-size aggregates can be observed. It is difficult to estimate an average particle size, but it seems to be sub-micrometric, with a size distribution centred at around 600 nm. The samples prepared by citrate chemistry happen to be more dispersed than those prepared by the ceramic route, so it is concluded that the morphology tightly depends on the synthesis route. Powders obtained by ceramic methods show well-crystallized pseudo-cubic particles having sharp facets, whereas those obtained *via* sol–gel present oblong-spherical shapes. The polymeric organic resin resulted in an ununiform carbon foam wherein the crystallites are embedded, as shown in Fig. 6b and c. The appearance of this “skin” is glassier after treatment with ammonia flux. Hypothetically, an improvement in the rate of lithium de-/intercalation reactions could be projected by tuning crystallite size and morphology by the sol–gel route, for what further synthesis optimizing studies should be performed. Fe and P elements, together with carbon from the polymeric coating determined by EDX confirmed a 1 : 0.98 ratio of Fe/P for both U-LFP and N-LFP compounds.

### Electrochemical properties

**Fig. 7:**
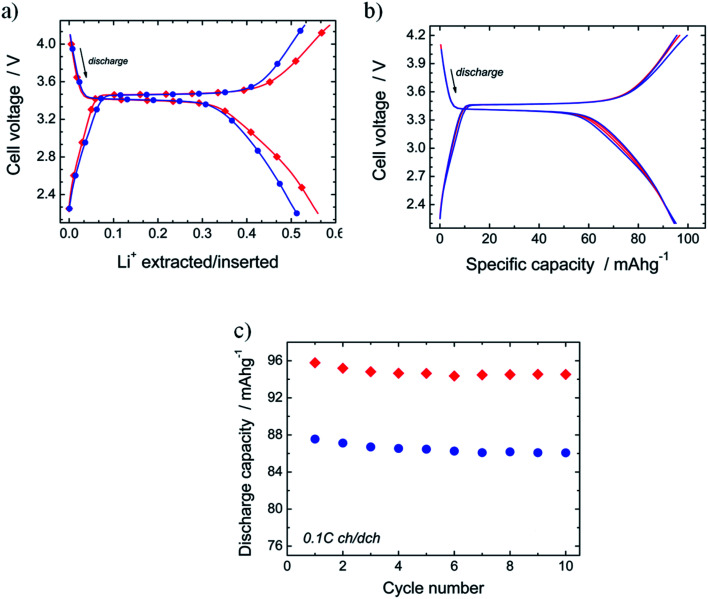
(a) Charge/discharge profile of N-LFP (red diamonds) and U-LFP (blue circles) registered at 0.1C (17 mA g^−1^). (b) Charge/discharge curves of several cycles (2^nd^, 4^th^, 7^th^ and 10^th^) recorded during the cycling of Li ‖ N-LFP cell at 0.1C rate, and (c) evolution of discharge capacity *vs.* cycle number for N-LFP (red diamonds) and U-LFP (blue circles).

The electrochemical performance of N-LFP as cathodic material was evaluated in a so-called half-cell, namely a lithium-cathode configuration. In Fig. 7a, the charge/discharge voltage profiles of N-LFP and U-LFP, registered at 0.1C rate (17.5 mA g^−1^), are exhibited. As can be observed, both curves show a similar profile shape. It presents a flat plateau, which is followed by a sloping variation of the voltage as the ΔLi^+^ de-/intercalation increases. This curve profile is commonly reported for LFP-based cathodes.^16,63,64^ The values of the redox potential for the plateau are identical for both olivines; they are 3.47 V *vs.* Li^+^/Li in charge and 3.40 V in discharge. This result shows that voltage is not modified by N-doping.

The theoretical^43^ and experimental^29,46^ studies also show that the average intercalation voltage ascribed to Fe^2+^/Fe^3+^ redox couple for nitrided and pure LiFePO_4_ are very close together. The reversible electrochemical process of lithium de-/insertion from/into LFP cathodes occurs according to the following reaction:LiFePO_4_ ⇔ Li_1−*x*_FePO_4_ + *x*(Li^+^ + e^−^)

In discharge, the amount of lithium inserted in the plateau is similar in both cases (ΔLi^+^ = 0.31; *i.e.*, discharge capacity *Q*_d_ = 53 mA h g^−1^). At the end of the discharge, ΔLi^+^ is higher for nitrided N-LFP (ΔLi^+^ = 0.59, *Q* = 95 mA h g^−1^) than for unnitrided U-LFP (ΔLi^+^ = 0.51, *Q* = 87 mA h g^−1^). For the determination of these values, several cells have been tested for each sample; in all cases, the capacity drain for nitrided-LFP cells was higher. The moderate values of the drained capacity for the studied samples are close to that reported for uncoated-LFP^5,16,17,19^ or samples for which the carbonaceous materials of the LFP/C composite have not formed an efficient coating.^65,66^ The effect of residual carbon on LiFePO_4_ properties is a thoroughly-studied topic,^39,41^ and it is well-known that carbon content should be lower than 10% and carbon coating layers of 3–4 nm are optimum.

In order to test the effect of the N-doping on cycling performances, ten galvanostatic cycles at low current (0.1C) in the potential range from 2.2 V to 4.2 V were carried out. Fig. 7b shows the charge/discharge curves registered for several cycles of the Li ‖ N-LFP cell. It is observed that all curves are almost identical. This result demonstrates the good cyclability of the synthesized nitrided N-LFP sample, at least in the few first cycles. In Fig. 7c, the evolution of the discharge capacity *vs.* cycle number for N-LFP (diamonds) and U-LFP (circles) is compared. The capacity retention after the ten cycles, determined as *Q*_Rt10_ = *Q*_10cycle_ × 100/*Q*_1cycle_, is high and very close for both samples: *Q*_Rt10_ = 98.7% for N-LFP, and 99.0% for U-LFP, as generally described for bulk LFP cathodes.^16,24,25,28^ As the reversible capacity of the nitrided sample (*Q*_rev_ ∼ 95 mA h g^−1^) is slightly higher than that of U-LFP (*Q*_rev_ ∼ 86 mA h g^−1^), it can be concluded that doping with nitrogen seems to improve the cycling performances.

**Fig. 8:**
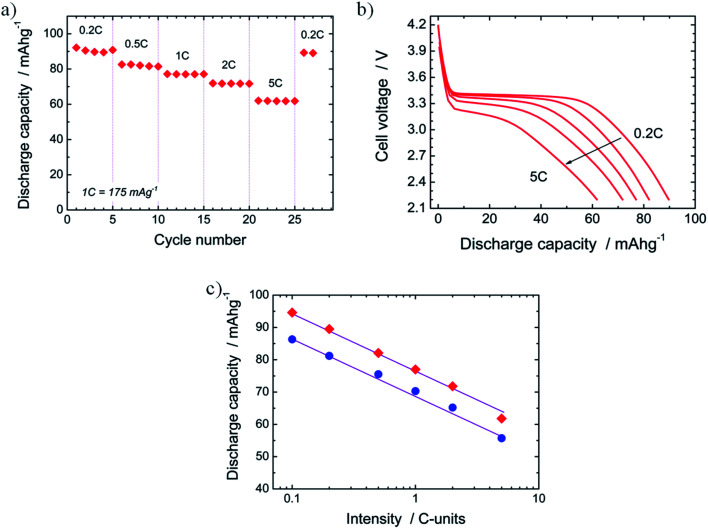
(a) Capacity at different discharge rates. (b) Discharge curve for each rate tested. (c) Evolution of discharge capacity *vs.* intensity current (in logarithmic scale) for N-LFP (red diamonds) and U-LFP (blue circles) registered in the voltage range from 2.2 to 4.2 V at 25 °C.

The performance of novel electrode materials for batteries against an increased discharge current is typically assessed when evaluating new electrode candidates. This study provides valuable information about the power that the material under review can deliver. In the present work, we have investigated the rate capability of N-LFP. In Fig. 8a, its capacity at different discharge rates is plotted. It is observed that the capacity decreases from *Q*_d_ = 89.5 mA h g^−1^ at 0.2C (35 mA g^−1^) to 61.8 mA h g^−1^ at 5C (875 mA g^−1^). In other words, N-LFP is capable of retaining 69% of its 0.2C capacity when the discharge intensity current is raised by a factor of 25. The capacity loss on increasing the discharge rate has been widely reported for LFP-based electrodes, especially for carbon-uncoated LFP samples.^40,64^ This behaviour is explained by kinetic limitations of Li^+^-insertion process due to the low electronic conductivity of the LFP^27,67,68^ and the slow diffusion of Li^+^ ions into the 1D-channels of the olivine structure.^27,68^ When the current was lowered to 0.2C after the rate capability study, it was observed that the 0.2C capacity was regained (*Q*_d_ = 89 mA h g^−1^). This confirms that the capacity loss is a reversible process due to kinetic limitations instead of irreversible changes in the structure of the N-LFP sample. Five cycles were performed for each tested current. In all cases, the discharge capacity remained almost unchanged on cycling. This is a remarkable result, as it shows that a good cyclability of the N-LFP is preserved even at high discharge currents (up to 5C). A discharge curve for each tested current rate is shown in Fig. 8b. Besides the aforementioned decrease in the capacity, an abatement of the plateau voltage from 3.40 V, at 0.2C, to 3.13 V, at 5C, was observed. Moreover, the flat plateau profile becomes sloping on increasing the discharge rate. The decrease in the plateau voltage and the changes in the discharge profile shapes can be explained by the internal resistance drop^62^ and the increase of the cell polarization when the current is raised.^26^ Both factors are controlled by the slow Li^+^-ion diffusivity during the charge/discharge steps from/into the bulk of the LFP-olivine^27,68^ and the low electrical conductivity of the olivine-LFP.^27,67–69^ A similar behaviour was observed for the U-LFP sample. The rate capability of N-LFP and U-LFP were also compared. In Fig. 8c, the evolution of discharge capacity *vs.* log(intensity) registered for the two samples is shown. For every rate, the capacity of N-LFP is systematically higher than that of U-LFP. When the evolution of *Q*_d_ is examined, a linear decrease of capacity on increasing the rate is observed. The slopes of both straight lines are very close. They are −18.9(7) for N-LFP and −17.5(8) for U-LFP. When the slopes are normalized – where slope = slope × 100/(*Q*_d_ at 0.2C) – they behave analogously, −20.0% for N-LFP and −20.3% for U-LFP. When all the rate capability results are considered, it can be concluded that the rate performances of N-LFP, as it was deduced from the cycling behaviour, are better in spite that *Q*_d_ decreases similarly on increasing the rate; nevertheless, the discharge capacity of the N-LFP is always higher than those of unnitrided LiFePO_4_.

Regarding the determined features of the crystal structure, the shorter Li–N distances observed when N is replacing O2 would actually suggest a hindrance of the Li diffusion, but this is not the case due to a polarisation on the N atom towards P and a consequent increase in the covalence of the framework. This causes the Li^+^ site and percolation energies to lower upon nitridation and is also reflected with a better performance in capacity and cyclability of the battery. It should be mentioned that the theoretical capacity is not reached by any of the samples (neither U-LPF nor N-LPF), owing to the uncoated nature of the grains; coating modifies the electrical conductivity of the samples and this parameter is probably altered by N doping since it involves partial oxidation of Fe^2+^ to Fe^3+^ and hence an improvement of the electron hopping throughout the structure. Therefore, despite the shorter Li–N/O2 distances, the enhancement of the ionic and electronic conductivity is the driving force for the better performance of the nitrided sample.

## Conclusions

We have prepared a new oxynitride with olivine structure by ammonolysis of the parent LiFePO_4_ oxide. Neutron powder diffraction data allowed us to refine the nitrogen content and its distribution over the anionic sublattice. N-atoms are shown to be located at random at O2 crystallographic positions. The crystallographic formula, Li_0.94_FePO_3.84_N_0.16_, suggests an average oxidation state of 2.2 + for Fe cations, assuming full electron transfer to O^2−^ and N^3−^ anions since Li stoichiometry is preserved. Olivine particle sizes and their distributions proved to rely on the synthesis procedures used to obtain the samples: (i) even submicrometric LFP crystallites were obtained by sol–gel approaches (citrate chemistry), while (ii) ceramic methods led to micrometric-sized materials with uneven particle size distribution. The best sample was obtained from the nitridation of precursors derived from metal citrates. It is also noteworthy that the ceramic reactions under NH_3_ atmosphere also proceed at mild temperatures (650 °C) and short times of about 3 hours. The decomposition of the organic resins in an inert or reducing atmosphere prevents the combustion, in such a way that an important amount of carbon is present in the samples. The different electrochemical tests have shown that: (i) the LFP working voltage does not change with the nitridation, and (ii) the specific capacity, cyclability and rate capability result to be systematically higher for the nitrided LFP-sample regarding the unnitrided precursor. The differences are small, but despite the relatively low concentration of nitrogen structurally incorporated into the sample, a significant influence of the nature of the anion sublattice on the macroscopic behaviour is observed.

## Author contributions

Sergio Federico Mayer: software, validation, writing – original draft preparation, writing – review & editing, visualization. Cristina de la Calle: validation, investigation, writing – original draft preparation. José Manuel Amarilla: conceptualization, methodology, validation, resources, writing – original draft preparation, visualization. José Antonio Alonso: conceptualization, methodology, software, validation, resources, writing – original draft preparation, visualization, project administration, funding acquisition.

## Conflicts of interest

There are no conflicts to declare.

## Supplementary Material

RA-012-D1RA07574H-s001

RA-012-D1RA07574H-s002

RA-012-D1RA07574H-s003
