# Supplementary material for: Nitridation effect on lithium iron phosphate cathode for rechargeable batteries
Source: RSC Adv. 2022 Jan 28;12(6):3696–707. doi: 10.1039/d1ra07574h (PMC8979366; doi:10.1039/d1ra07574h)
Supplement: RA-012-D1RA07574H-s001 [file RA-012-D1RA07574H-s001.pdf]

Electronic Supplementary Material (ESI) for RSC Advances.
This journal is © The Royal Society of Chemistry 2022

Caption for the animated figure (Supplementary File). Animation of the percolation pathway for N-LFP along the **b** direction, corresponding to the one-dimensional percolation path of lowest activation energy, $Ea_{\mathbf{b}}$ = 0.91 eV. Li1 species is located within the percolation pathway, here presented as a yellow isosurface. The PO_4^- groups are presented as violet tetrahedra, while the O-coordinated Fe species are exhibited as brown, strongly-distorted octahedra. Li ions are presented as green spheres of 0.94 occupancy. Oxygen atoms are shown as red spheres, while the substituting N species in violet partly fills the sites mostly occupied by O2.
