# Supplementary figures and images for: Nitridation effect on lithium iron phosphate cathode for rechargeable batteries
Source: RSC Adv. 2022 Jan 28;12(6):3696–707. doi: 10.1039/d1ra07574h (PMC8979366; doi:10.1039/d1ra07574h)

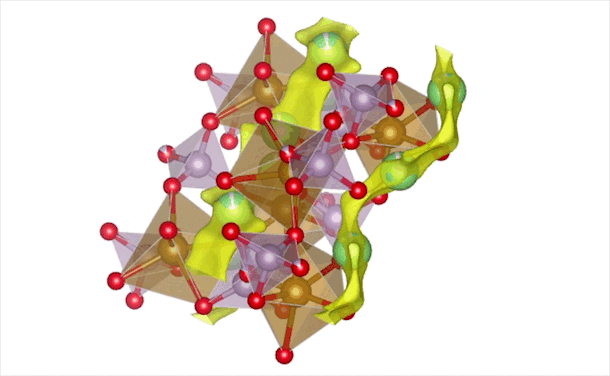

Supplement: RA-012-D1RA07574H-s002 [file RA-012-D1RA07574H-s002.gif]
